# Identification of giant Mimivirus protein functions using RNA interference

**DOI:** 10.3389/fmicb.2015.00345

**Published:** 2015-04-28

**Authors:** Haitham Sobhy, Bernard La Scola, Isabelle Pagnier, Didier Raoult, Philippe Colson

**Affiliations:** ^1^URMITE UM63 CNRS 7278 IRD 198 INSERM U1095Faculté de Médecine, Aix-Marseille University, Marseille, France; ^2^IHU Méditerranée Infection, Pôle des Maladies Infectieuses et Tropicales Clinique et Biologique, Fédération de Bactériologie-Hygiène-Virologie, Centre Hospitalo-Universitaire Timone, Assistance Publique-Hôpitaux de MarseilleMarseille, France

**Keywords:** Mimivirus, giant virus, Megavirales, fiber, short interfering RNA, RNA interference, nucleocytoplasmic large DNA virus

## Abstract

Genomic analysis of giant viruses, such as Mimivirus, has revealed that more than half of the putative genes have no known functions (ORFans). We knocked down Mimivirus genes using short interfering RNA as a proof of concept to determine the functions of giant virus ORFans. As fibers are easy to observe, we targeted a gene encoding a protein absent in a Mimivirus mutant devoid of fibers as well as three genes encoding products identified in a protein concentrate of fibers, including one ORFan and one gene of unknown function. We found that knocking down these four genes was associated with depletion or modification of the fibers. Our strategy of silencing ORFan genes in giant viruses opens a way to identify its complete gene repertoire and may clarify the role of these genes, differentiating between junk DNA and truly used genes. Using this strategy, we were able to annotate four proteins in Mimivirus and 30 homologous proteins in other giant viruses. In addition, we were able to annotate >500 proteins from cellular organisms and 100 from metagenomic databases.

## Introduction

*Acanthamoeba polyphaga* mimivirus was the first member discovered of the viral family *Mimiviridae*, which encompasses viruses that infect *Acanthamoeba* sp. (La Scola et al., 2003; Raoult et al., 2004). Subsequently, dozens of Mimivirus relatives have been isolated from environmental samples and, more recently, from humans (La Scola et al., 2008; Boyer et al., 2009; Fischer et al., 2010; Arslan et al., 2011; Yoosuf et al., 2012; Saadi et al., 2013a,b). Other viruses that infect protozoa were also subsequently discovered, including marseilleviruses (Boyer et al., 2009; Boughalmi et al., 2013a,b; Aherfi et al., 2014), pandoraviruses (Philippe et al., 2013) and *Pithovirus sibericum* (Legendre et al., 2014). Mimiviruses have been linked, along with the marseilleviruses, to the nucleocytoplasmic large DNA viruses (NCLDVs), which were recently proposed to be unified into a new viral order named the “*Megavirales*” (Colson et al., 2013). These giant viruses have raised considerable interest in the field of evolutionary biology because of their unexpectedly large size, as well as the fact that they contain genes encoding functions previously believed to be in the domain of cellular organisms, such as aminoacyl-tRNA synthetases or translations factors. They have challenged the definition of a virus (Raoult et al., 2004; Moreira and Brochier-Armanet, 2008; Raoult and Forterre, 2008; Forterre, 2010).

The genomes of *Megavirales* members contain a large number of predicted genes annotated either as hypothetical proteins or ORFan genes, i.e., genes without homologs in sequence databases (Raoult et al., 2004; Boyer et al., 2010). For example, genes encoding hypothetical proteins occupy more than 50% of the Mimivirus genome. The functions of these genes are not known. To date, the functions of only a few Mimivirus proteins have been studied experimentally, including amino-acyl-tRNA synthetases (Abergel et al., 2005, 2007) and proteins involved in sugar biosynthesis (Piacente et al., 2012). Thus, the large majority of Mimivirus genes have no known function and make up a ‘functional dark matter.’

The Mimivirus capsid, which is approximately 500 nm in size, is covered by a dense layer of fibers. These viral fibers are approximately 125–140 nm in length and approximately 1.4 nm in diameter and consist of a soft shaft and a globular shaped head (Xiao et al., 2009; Klose et al., 2010; Kuznetsov et al., 2010). Clusters of 3–4 fibers were found to be linked via a disk shaped base. They are highly glycosylated, antigenic, and resistant to protease and collagenase treatment (Xiao et al., 2009; Boyer et al., 2011). A putative GMC-type oxidoreductase (R135), and two hypothetical proteins (L725, which is the product of an ORFan, and L829) were identified in purified fibers by gel electrophoresis coupled with matrix-assisted laser desorption/ionization mass spectrometry (MALDI MS; Boyer et al., 2011). Sub-culturing Mimivirus 150 times on germ-free amoebae led to the emergence of a mutant “M4” strain lacking fibers, and with a genome reduced by 16% and missing 150 genes (Boyer et al., 2011). Comparative proteomics of M4 and the original Mimivirus strain showed a deletion of the R135 and L829 proteins, as well as of the R856 protein (Boyer et al., 2011), which belongs to the group of tetratricopeptide repeat (TPR) containing proteins previously involved in virus–host interactions (Jeshtadi et al., 2010). In addition, nine proteins have been proposed to be involved in sugar biosynthesis and fiber formation (Piacente et al., 2012; Supplementary Table S1).

**FIGURE 1:**
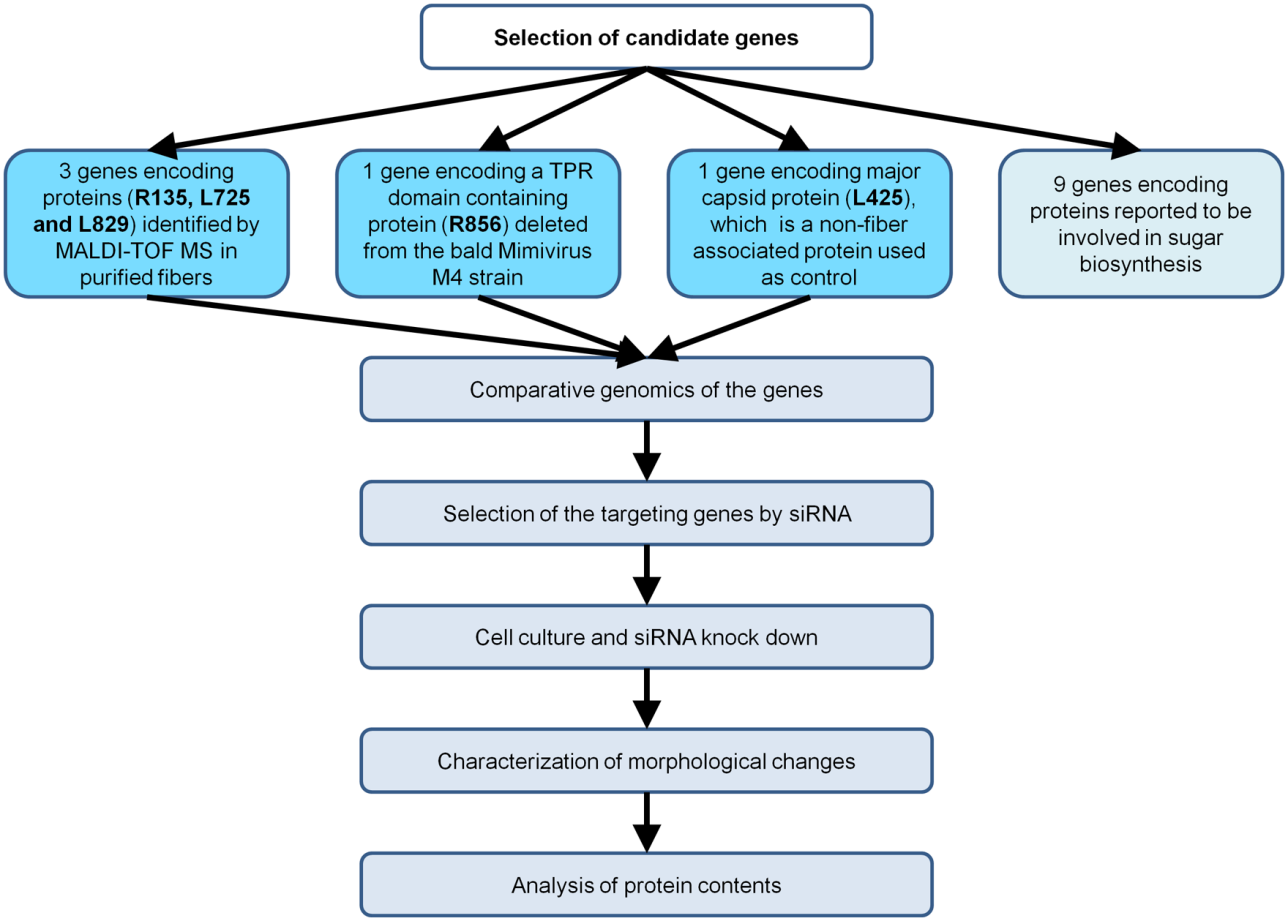
**Flow chart of the strategy implemented to characterize Mimivirus gene function using siRNA**.

Here, we aimed to apply RNA interference (RNAi) to the identification of the function of Mimivirus proteins. We targeted four Mimivirus genes associated with fiber formation, as fibers can be easily observed by electron microscopy (**Figure 1**).

## Materials and Methods

### Cell Culture, siRNA, and Identification of Morphological Changes

#### Targeted Genes and siRNA

We targeted the Mimivirus genes R135, L725, L829, and R856 using short interfering RNA (siRNA). These genes were either identified in purified fibers or deleted in the M4 strain (Boyer et al., 2011; **Figure 1**). Regarding the negative controls, we used Mimivirus virions in absence of treatment with siRNAs, and also targeted the L425 gene, which is known to be expressed and encodes the major capsid protein that composes the Mimivirus shell located just beneath the fiber layer. The aim of this control was to ensure for the absence of possible non-specific, artefactual effect of siRNAs or lipid carrier on fibers.

#### Cell Culture and RNAi

A culture of *A. polyphaga* in 10 ml of PYG medium (5e5 amoeba/mL) was seeded for 24–48 h. Then, 100 μl of Lipofectamine RNAiMAX (Invitrogen, USA) and 0.25 μg of duplex siRNA (designed and purchased from Invitrogen; for sequences, see Supplementary Table S2) were used according to the manufacturer’s instructions. To improve the siRNA specificity, we used duplex siRNA and we checked for specific and non-specific appariements by performing BLASTn searches against Mimivirus genes and GenBank. One ml of Mimivirus preparation (≈1e^6^ viruses) was added to the culture, and incubated for 11 h at 32°C. *A. polyphaga* were harvested by centrifugation (500 *g* for 10 min) and analyzed by electron microscopy. For protein analysis, four 10-ml flasks of Mimivirus culture incubated for 24 h at 32°C were used. Cells were completely lysed. Then, the culture medium was centrifuged at 500 *g* for 10 min, and the supernatant was filtered through a 1.2-μm filter to eliminate *A. polyphaga* cell debris. The Mimivirus pellet, obtained by centrifugation of the medium at 12,000 *g* for 15 min, was washed twice with phosphate buffer serum (PBS), and the purified viruses were used in further investigations. For the negative control gene, the same experimental procedures were applied to the L425 gene encoding capsid protein.

#### Electron Microscopy

The preparation of samples for electron microscopy was previously described (Boyer et al., 2011). Briefly, samples were fixed with glutaraldehyde (2.5%) and cacodylate buffer (0.1 M), cut into 70-nm sections using an ultramicrotome (UC7; Leica), collected on 400-mesh nickel grids with formvar carbon, and stained for electron microscopy (FCF-400-Ni, Electron Microscopy Sciences). The samples were then viewed with a Philips electron microscope (Morgagni 268D) at 80 keV. Cross sections of all pictures that were selected for the analysis were positioned at the middle of the virions and characterized by a dense cluster (black mass).

### Analysis of Protein Content of Knocked Down Viruses

#### Antibodies Preparation

Fibers were purified from the virus as previously described for vaccinia virus (Jensen et al., 1996). Previous analysis of the fiber by 2D-gel coupled with MALDI-TOF MS (Boyer et al., 2011) revealed that three proteins (R135, L725, and L829) were associated with Mimivirus fibers. For anti-L725 antibodies, L725 protein fused with thioredoxin was expressed in *Escherichia coli* and purified using ÄKTA avant 25 (GE Healthcare, USA). Purified Mimivirus virions, fibers, and L725 protein were injected into mice to obtain anti-L725 polyclonal antibodies, as previously described (Boyer et al., 2011).

#### Immunogold Labeling

Grids were immersed in NH_4_Cl (50 mM) diluted in PBS three times for 5 min, washed in PBS for 5 min, and then immersed twice in blocking buffer (1% normal goat serum (NGS), 1% bovine serum albumin (BSA), and 0.2% Tween 20 diluted in PBS; 2 × 10 min). The grids were incubated with anti-fiber polyclonal antibody that was diluted 1:100 in blocking buffer overnight at 4°C. After four 10-min washes, the grids were incubated for 90 min in biotin (Beckman Coulter, USA) that was diluted 1:100 in blocking buffer. Then, the grids were washed with 0.1% BSA-PBS (4 × 5 min) and then in 0.01% cold water fish skin (CWFS) gelatin-PBS (3 × 10 min), and incubated with streptavidin (labeled by 10-nm gold nano-particles; Aurion, The Netherlands) that was diluted 1:100 in 0.01% CWFS gelatin-PBS for 90 min and washed with PBS. After incubating with PBS-glutaraldehyde 2.5% for 15 min, the grids were washed with PBS (2 × 10 min) and distilled water for 10 min. Finally, the grids were contrasted by adding uranyl acetate for 20 min, immersed in water 60 times, and analyzed using electron microscopy. The number of gold particles that were bound to fibers in each image was counted. The experiments included several steps that were performed on successive days. For each step, we used Mimivirus treated without siRNA with the same experimental conditions as the siRNA-treated Mimivirus as negative control.

#### Proteomic Analysis

All proteomic analysis (sample preparation, 1D and 2D gel electrophoresis, silver staining, and western blotting) was performed as previously described (Azza et al., 2009). Briefly, Mimivirus was solubilized in 40 mM Tris-HCl, pH 7.5, supplemented with 2% (wt/vol) sodium dodecyl sulfate (SDS; Sigma–Aldrich) and 60 mM dithiothreitol (DTT), followed by 5 min of heating at 95°C. The insoluble fraction was removed by centrifugation (12,000x *g*, 4°C, 10 min), and soluble proteins were precipitated using a PlusOne 2-D Clean-Up kit (GE Healthcare, USA) to remove SDS. The final pellet was re-suspended in solubilization buffer [7 M urea, 2 M thiourea, 4% (wt/vol) 3-[(3-cholamidopropyl)-dimethylammonio]-1-propanesulfonate (CHAPS)] and stored at -80°C until use. The protein concentration was measured by Bradford assay (Bio-Rad, USA). Immobiline DryStrips (13 cm, pH 3–10; GE Healthcare) were rehydrated overnight using 250 μl rehydration buffer [8 M urea, 2% (w/v) CHAPS, 60 mM DTT, 0,5% (v/v) IPG buffer (GE Healthcare)] containing 20 μg of solubilized Mimivirus proteins and isoelectric focusing (IEF) was carried out according to the manufacturer’s protocol (IPGphor II, GE Healthcare). Before the second dimension electrophoresis was performed, strips were equilibrated twice in 5 ml equilibration buffer [30% (v/v) glycerol, 3% (w/v) SDS, 6 M urea, 50 mM Tris-HCl, bromophenol blue, pH 8.8] for 15 min. This buffer was supplemented with 65 mM DTT for the first equilibration and with 100 mM iodoacetamide for the second one. The strips were then embedded in 0.5% agarose and the proteins resolved by 10% SDS-PAGE (Protean II XL, Bio-Rad). Gels were stained either with silver or transferred onto nitrocellulose membranes for western blot analysis using anti-Mimivirus, anti-L725 or anti-fiber primary polyclonal antibodies. Then, the membrane was washed three times with PBS-Tween and probed for 2 h with horseradish peroxidase-conjugated goat anti-mouse secondary antibodies.

### Comparative Genomics and Phylogenetic Tree Reconstruction

Protein sequences of mimiviruses were retrieved from the NCBI GenBank non-redundant protein sequence database (nr) (http://blast.ncbi.nlm.nih.gov/Blast.cgi). BLASTp searches were performed with 0.01 as the *e*-value cutoff. The best hits were collected and aligned using ClustalW (Larkin et al., 2007). The multiple sequence alignments were trimmed by Gblock (Castresana, 2000). Phylogenetic tree reconstructions were performed using the Maximum likelihood method of the FastTree tool with default parameters (Price et al., 2010).

### PCR Testing

The presence or absence of Mimivirus genes in the purified viral solution was determined by qPCR. The most conserved sites were identified, and universal primers and probes were designed using the Gemi tool (Sobhy and Colson, 2012; Supplementary Table S3). The 25 μl-real-time PCR mixture contained 5 μl of extracted DNA, 12.5 μl qPCR Mastermix (Eurogentec, Belgium), 0.5 μl of each primer (10 nmol/μl; Eurogentec), and 0.5 μl probe (3 nmol/μl; Applied Biosystems UK). The PCR thermal cycling conditions were: a hold at 50°C for 2 min, a hold at 95°C for 5 min, and then 45 cycles of 30 s at 95°C then 1 min at 60°C.

## Results

### Consequences of Silencing Targeted Mimivirus Genes on Fiber Formation

**FIGURE 2:**
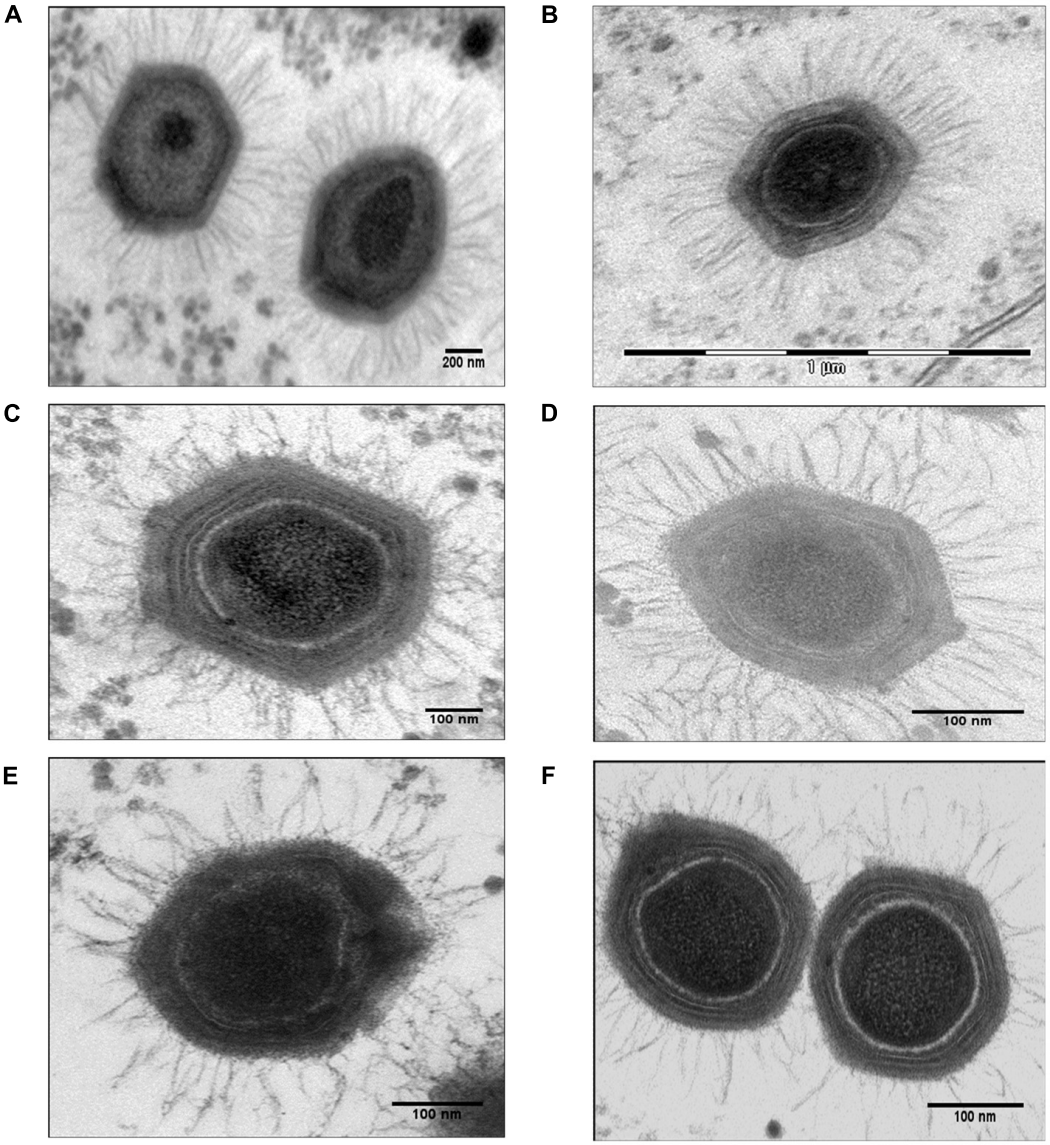
**Electron micrographs of control Mimivirus and Mimivirus after knocking down genes encoding fiber associated proteins (FAPs). (A)** Control, **(B)** Control si-L425 Mimivirus (meaning Mimvirus treated with siRNA targeting the L425 gene); **(C)** si-R856 Mimivirus, **(D)** si-L725 Mimivirus, **(E)** si-L829 Mimivirus, and **(F)** si-R135 Mimivirus. The pictures were taken for virions within *Acanthamoeba polyphaga* host. See Supplementary Figures S1–S6 for additional figures.

**FIGURE 3:**
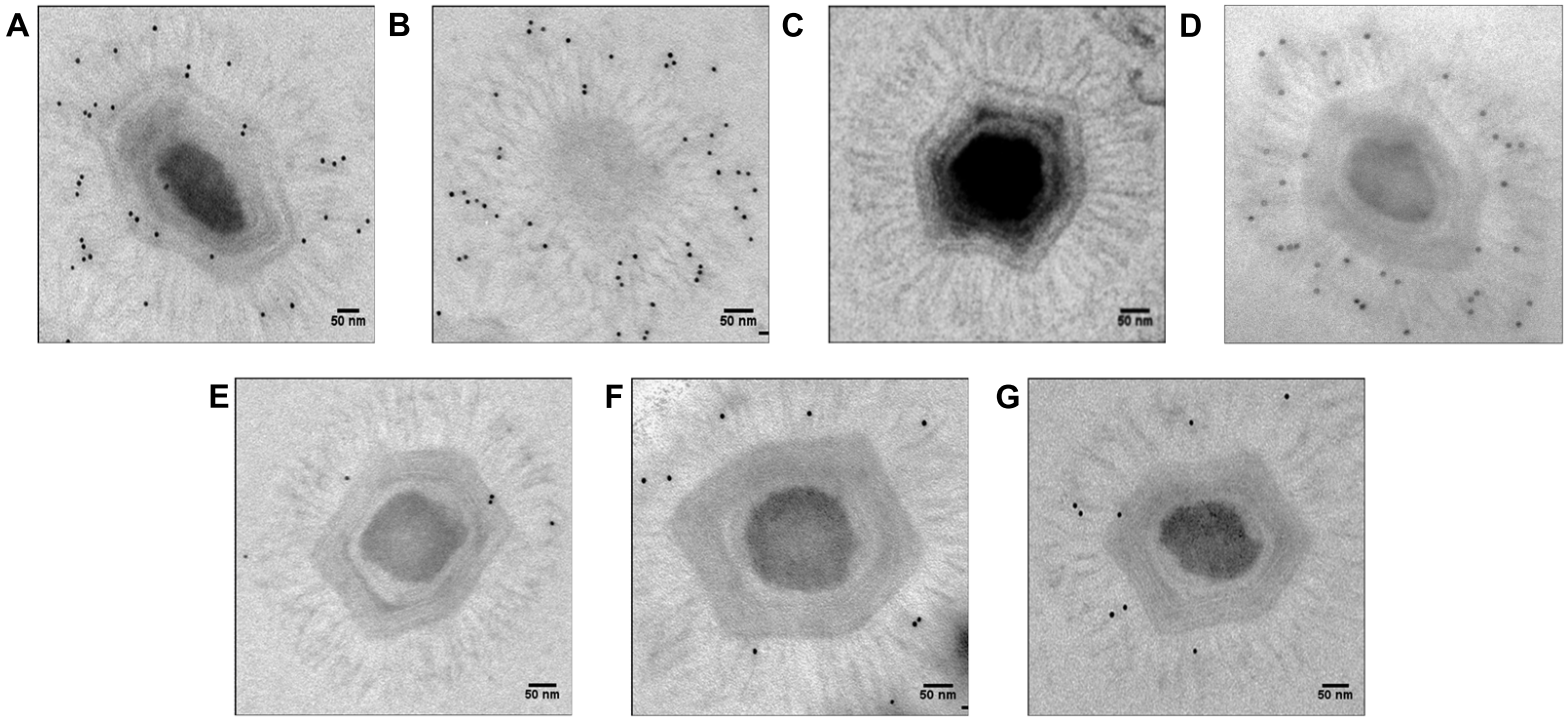
**Electron microscopy with immunogold targeting of Mimivirus fiber proteins using anti-fiber antibodies (1:100). (A)** Positive control, **(B)** Positive control (view from one of the capsid vertices), **(C)**. Negative control with only secondary, not primary, antibodies, **(D)** si-L425 Mimivirus (meaning Mimvirus treated with siRNA targeting the L425 gene), **(E)** si-L725 Mimivirus, **(F)** si-L829 Mimivirus, and **(G)** si-R856 Mimivirus. The pictures were taken for virions outside *A. polyphaga* host.

We knocked down the Mimivirus genes encoding the R135, L725, L829, R856, and L425 proteins using siRNA. We then compared the fibers from the viruses produced under these conditions to those from control viruses produced in the absence of siRNA or treated with siRNA targeting non-fiber associated proteins (L425), searching for any abnormal feature of the fibers, such as short, prone (procumbent), or non-stretched and curved fibers (**Table 1**; **Figure 2**; Supplementary Figures S1–S6). To measure length ratio for silenced versus control viruses, we selected 4–8 viruses that harbored ≥30 fibers and measured the lengths of the fibers in each condition (**Table 2**). To determine protein contribution in fiber formation, we counted the number of gold particle conjugated with anti-fiber antibodies, hence to Mimivirus fibers (**Table 3**; **Figure 3**).

**Table 1:** Number of viruses with fibers with normal or abnormal features.

Silenced gene	Total number of viruses observed	Abnormality %	Average abnormality %	Number of viruses with short fibers (%)	Number of viruses with sparse/curved fibers (%)
Control	91	0	0	0 (0)	0 (0)
si-L425 (control)	90	0	0	0 (0)	0 (0)
si-R135	123	16–54	35	20 (16)	47 (38)
si-L725	52	21–71	46	11 (21)	26 (50)
si-L829	90	13–49	31	12 (13)	32 (36)
si-R856	101	58–63	61	59 (58)	5 (5)

*Normal fibers are those with classical shape and >100 nm-long, dense and stretched fibers. Sparse/curved fibers are fibers that are not as thick or dense as the normal or non-stretched fibers. Short fibers are those with a length <100 nm. The percentage of abnormality indicates the percentage of viruses with either short or non-stretched and sparse fibers. si-, virus after silencing the target gene. See also Supplementary Figures S1–S6.*

**Table 2:** Length of the Mimivirus fibers according to each siRNA experimental condition.

Virus	Number of viruses observed	Number of fibers	Mean length of fibers ± standard deviation	Relative length of fibers compared to control (%; silenced / control fiber ^∗^100)	*p*-value^1^
Control	8	30	131.4 ± 23.8	100.0	–
si-R135	6	34	115.0 ± 27.5	87.5	0.0138
si-L725	4	31	110.8 ± 26.0	84.4	0.0021
si-L829	7	33	91.8 ± 25.0	69.9	<1e-6
si-R856	8	29	47.8 ± 18.8	36.4	<1e-6

*^1^*P*-values were calculated using the ANOVA test (http://www.openepi.com). si-, virus after silencing the target gene. See also Supplementary Figures S1–S6.*

**Table 3:** Number of gold nanoparticles per virus in immunogold.

Type	Number of viruses observed	Total number of gold particles	Mean number of gold particles per virus ± SD	Decrease in particles per virus (%)
Control 1 (no siRNA)	30	880	29 ± 7	0
si-L829	48	611	13 ± 6	57
si-R856	44	249	6 ± 3	81
si-L725	31	129	4 ± 3	86
Control 2 (no anti-fiber antibodies)	39	0	0 ± 0	100

*The percentage of reduction measures the drop or reduction in gold particles after knocking down each gene. si-, virus after silencing the target gene.*

#### Control Mimivirus Fibers

The average length of control fibers was 131 nm (**Table 2**; **Figures 2A** and **3A–C**, Supplementary Figure S1). Silencing the gene encoding the L425 protein did not affect fiber length or topology (**Figures 2B** and **3D**, Supplementary Figure S2).

#### Fibers after R856 Gene Silencing

Approximately 60% of si-R856 viruses (meaning viruses treated with an siRNA targeting the R856 gene) harbored abnormal or short fibers (**Table 1**; **Figure 2C**, Supplementary Figure S3). We observed that the average length of fibers from si-R856 viruses was 48 nm, which was 64% shorter than control viruses (*p* < 1e-6; **Table 2**). The number of gold particles bound to fibers was decreased by 81% after silencing R856 (**Table 3**; **Figure 3**).

#### Fibers after L725 Gene Silencing

Si-L725 viruses harbored approximately 50% abnormal curved fibers, which were 15% shorter than control fibers (**Tables 1** and **2**). The number of gold particles bound to fibers was decreased by 86% after silencing the L725 gene (**Table 3**; **Figures 2D** and **3E**; Supplementary Figure S4).

#### Fibers after L829 Gene Silencing

The fiber length of si-L829 viruses was 92 nm, which was 30% shorter than the control virus fibers (**Table 2**). In addition, the fiber layer was sparse in si-L829 viruses and the gold particle count was decreased by 57% (**Tables 1** and **3**; **Figures 2E** and **3F**; Supplementary Figure S5).

#### Fibers after R135 Gene Silencing

The fiber length of the si-R135 viruses was 12% shorter than the control and 38% of these fibers were curved (**Table 2**; **Figure 2F**, Supplementary Figure S6).

Additionally, Mimivirus is surrounded by fibers that are glycosylated, and usually there is a space surrounding the virion that separates the viral particle from the surrounding intracytoplasmic content of amoebas. This space can be observed clearly for all control viruses but not for silenced viruses. These findings indicate that (i) the presence of short or sparse fibers after siRNA treatment was due to the siRNAs, and not to lipid carrier or suboptimal experimental conditions, and that these siRNAs were specific to their targeted genes as we observed different characteristics of the Mimivirus fibers and their reactivity with antibodies depending on which gene was targeted by siRNAs; (ii) the R135, L725, L829, and R856 proteins are either principal elements of Mimivirus fibers or play a key role during fiber biosynthesis and can be functionally annotated as fiber associated proteins (FAPs); and (iii) the L725 and R856 proteins are major contributors to fiber formation.

### Consequences of Silencing Targeted Genes on Protein Content

**FIGURE 4:**
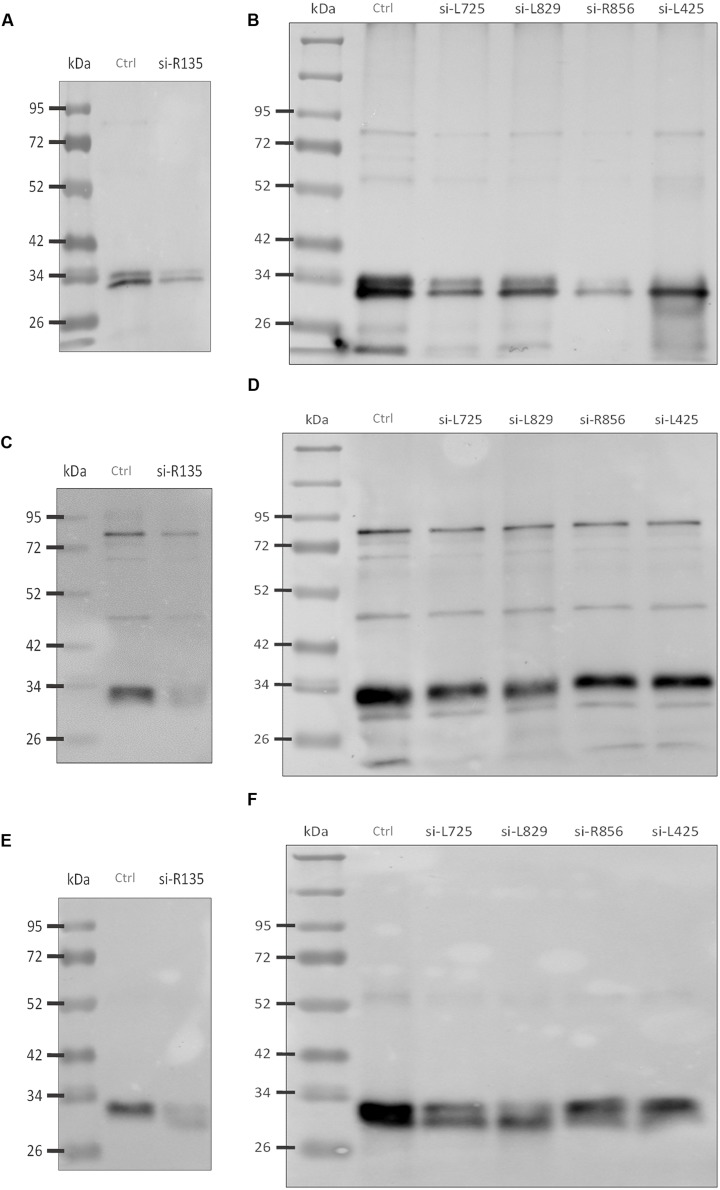
**Western blot analyses of fibers from silenced Mimivirus and control Mimivirus. (A)** Reduction in anti-Mimivirus fiber antibodies (1:1000) binding to fiber proteins of si-R135 Mimivirus and **(B)** Reduction in anti-fiber antibodies (1:1000) binding to fiber proteins of si-L725, si-L829, si-R856, and si-L425 Mimivirus, **(C)** Reduction in anti-Mimivirus antibodies (1:5000) binding to si-R135 Mimivirus, and **(D)** Reduction in anti-Mimivirus antibodies (1:5000) binding to si-L725, si-L829, si-R856, and si-L425 Mimivirus, **(E)** Reduction in anti-L725 antibodies (1:1000) binding to si-R135 Mimivirus, and **(F)** Reduction in anti-L725 antibodies (1:1000) binding to si-L725, si-L829, si-R856, and si-L425 Mimivirus. Figures correspond to different experiments. Ctrl indicates control Mimivirus; si- indicates the virus after silencing a target gene; molecular masses are indicated on the left; molecular masses for FAPs are as follows: L725: 27 kDa; R856: 40 kDa; L829: 50 kDa; and R135: 77 kDa.

**FIGURE 5:**
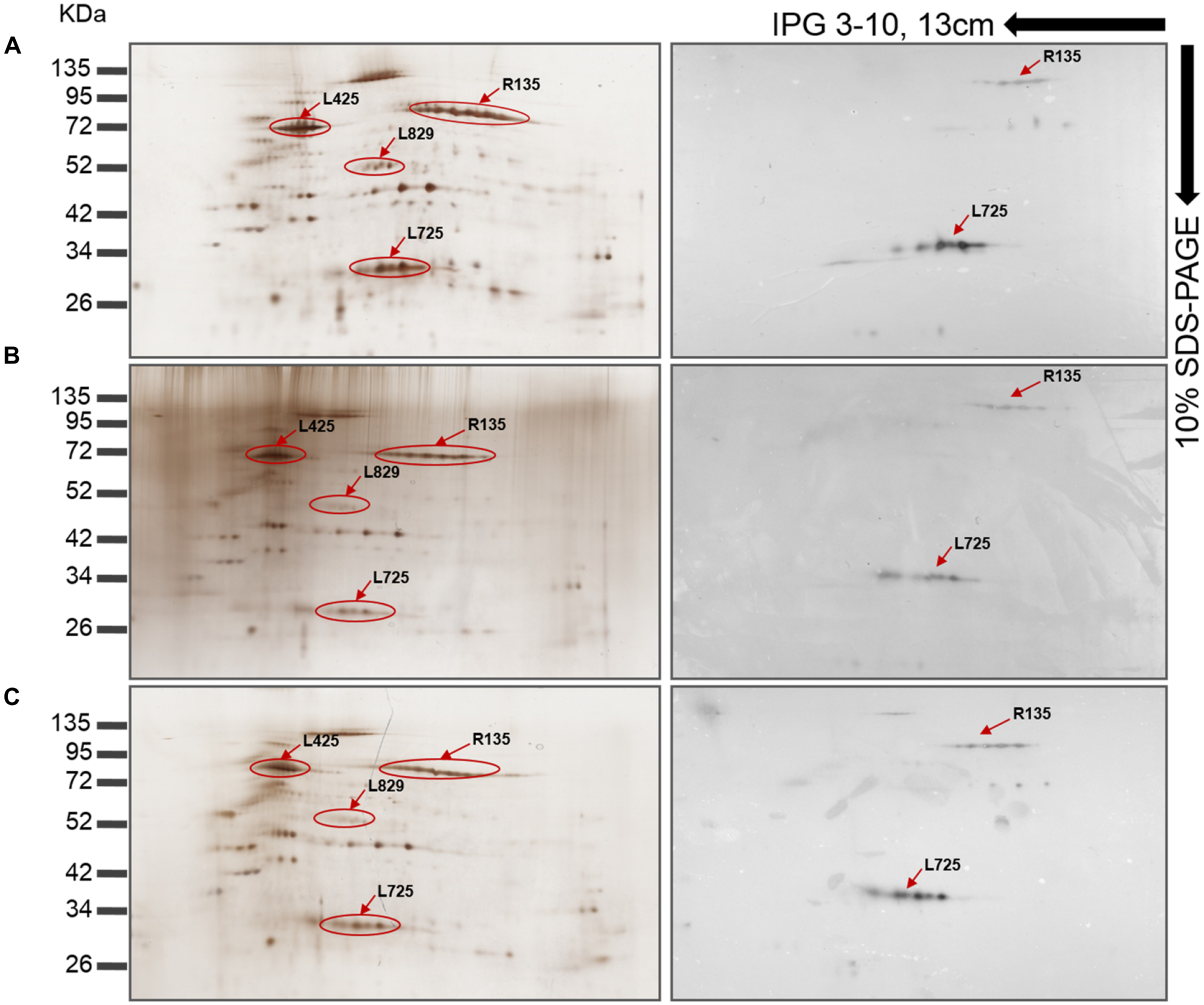
**Mimivirus protein profiles as shown by 2D-gel electrophoresis and western blot with anti-fiber antibodies (1:5000). (A)** Silver stained and western blotted gel electrophoresis of Mimivirus in the absence of siRNA. **(B)** Silver stained and western blotted gel electrophoresis of si-L829 Mimivirus. **(C)** Silver stained and western blotted gel electrophoresis of si-R856 Mimivirus. The decrease in spot intensity indicates the depletion of fiber proteins. Immunoreactive protein spots are shown using arrows and the locus names; the spots were previously identified in (Renesto et al., 2006; Boyer et al., 2011).

Western blot analyses were performed to validate these results and revealed that the reactivity of antibodies to FAPs was reduced against viruses whose genes were silenced compared to control viruses (**Figures 4A,B**). Thus, intensity for three bands that might correspond to R135, L725, and L829 molecular masses were reduced in silenced viruses. To confirm these results, nitrocellulose membranes were incubated with anti-Mimivirus (**Figures 4C,D**) and anti-L725 antibodies (**Figures 4E,F**), which showed reduced reactivities against silenced viruses, although some differences in reactivities were minor. These results suggest that the R135, L725, L829, and R856 proteins might be associated with Mimivirus fiber formation and changes observed in the Mimivirus fiber layer might be due to depletion of these proteins (i.e., post-transcriptional events). 2D-gel electrophoresis western blots performed for both si-L829 and si-R856 viruses also revealed a reduction in anti-fiber antibodies bound to the targeted FAPs compared to control viruses (**Figure 5**). Thus, knocking down the genes encoding R135, L725, L829, and R856 protein led to some decreases in the binding of anti-fiber antibodies to these proteins, which indicate that they may play a role in fiber formation. Taken together, TEM results, immunogold and proteomic analyses showed that R135, L725, L829, and R856 proteins can be functionally annotated as FAPs.

### Comparative Genomics and Protein Re-Annotation

We propose here that the R135, L725, L829, and R856 proteins can be annotated as Mimivirus FAPs, and their names can be abbreviated as FAP1, FAP2, FAP3, and FAP4, respectively.

Searching for sequence homology of the FAPs with proteins from giant virus *Megavirales* members and other organisms from public sequence databases revealed that these proteins are conserved in most of the giant viruses, but do not share sequence homology with any fiber or spike protein encoded by any virus, including adenoviruses (**Tables 4** and **5**; Supplementary Table S1; Figure S7). FAP2 (L725) is an ORFan only present in mimiviruses. FAP4 (R856) contains seven TPR domains and shares sequence similarity with hypothetical proteins encoded by archaea, bacteria, choanoflagellida, ciliophora, metazoa (including rotifera, cnidaria, and hydra), and from metagenomes, but not with any protein encoded by any virus (**Table 5**, Supplementary Table S1; Supplementary Figures S8–S10). FAP1 (R135) shares homology with oxidoreductases and hypothetical proteins encoded by *Acanthamoeba*, metazoa, fungi, and bacteria, including proteobacteria, as well as *P. sibericum*, and metagenomes. Finally, FAP3 (L829) is encoded by mimiviruses, marseilleviruses, *Pandoravirus* sp., and shares similarity with hypothetical proteins encoded by bacteria, and eukaryotes, including amoebozoa and fungi. Phylogenetic analyses indicate that FAP4 is widely distributed among environmental and aquatic species (Supplementary Figure S10). In addition, tree topologies suggest that FAP1 and FAP4 may have been subject to horizontal gene exchange with cellular organisms.

**Table 4:** The distribution of fibers and homologs to fiber associated proteins (FAPs) among viruses that infect *Acanthamoeba* sp. and were isolated in our laboratory or by other teams.

Virus	Fibers	R135 (FAP1)	L725 (FAP2)	L829 (FAP3)	R856 (FAP4)
APMV§	Yes	+ ^∗^	+ ^∗^	+ ^∗^	+ ^∗^
APMV-M4§	No	#	+ ^∗^	#	#
ACMaV§	Yes	+ ^∗^	+ ^∗^	+ ^∗^	+ ^∗^
APLenV§	Short	+	+	+	+
APMoV§	Yes	+	+	+	
Monv§	No	+	+	+	
Goul§	No	+	+	+	+
Crdo11§	Yes	+	+	+	
Crdo7§	Yes	+	+	+	
MegCV	Yes	+	+	+	
MarsV§	No			I	
LauV	No			I	
PsV	Yes			I	
PdV	Yes			I	
Pvs		I			
Sputnik§	Yes				

*(+) means detected as a hit in the NCBI BLASTp search, with an *e*-value <0.01 and identity >40%. (I) identity is 20–30%; (II) identity is 30–40%; ^∗^Reliable qPCR threshold cycle (Ct) values; #deleted gene; §the virus was isolated in our laboratory (see also Supplementary Figure S7).*

*APMV, *Acanthamoeba polyphaga* mimivirus; APMV-M4, *Acanthamoeba polyphaga* mimivirus isolate M4; ACMaV, *Acanthamoeba castellanii* mamavirus; APMoV, *Acanthamoeba polyphaga* moumouvirus; CroV, *Cafeteria roenbergensis* virus BV-PW1; APLenV, *Acanthamoeba polyphaga* lentillevirus; LauV, Lausannevirus; MarsV, Marseillevirus; MegCV, *Megavirus chiliensis*; Crdo11, Courdo11 virus; Crdo7, Ccourdo7 virus; Goul, Moumouvirus goulette; and Monv, Moumouvirus monve; PsV, *Pandoravirus salinus*; PdV, *Pandoravirus dulcis*; Pvs, *Pithovirus sibericum* (La Scola et al., 2008; Boyer et al., 2009, 2011; Fischer et al., 2010; Arslan et al., 2011; Colson et al., 2011; Yoosuf et al., 2012; Philippe et al., 2013).*

**Table 5:** Number of BLASTp hits corresponding to each Mimivirus fiber-associated protein.

	*Mimiviridae*^1^	Viruses	*Archaea*	*Bacteria*	*Eukaryota*	Metagenome^2^
R135 (FAP1)	12	0	0	415	71	88
L725 (FAP2)	5	0	0	0	0	0
L829 (FAP3)	14	0	0	18	4	0
R856 (FAP4)	2	0	7	174	304	20

*The numbers represent the number of hits per group of organisms using BLASTp against the NCBI or UniProt Archive (UniParc)^*2*^ sequence databases. For details, see Supplementary Table S1. L725 gene is an ORFan and conserved only in mimiviruses. FAP means fiber associated protein.*

*^1^Includes hits in mimiviruses and closely related viruses, including pandoraviruses and *Pithovirus sibericum*; ^2^Retrieved from BLASTp search against UniProt Archive (UniParc) database, which includes UniProt Metagenomic and Environmental Sequences (UniMES) database.*

Taken together, these data indicate that Mimivirus FAPs are divergent from proteins that are encoded by other viruses, including *Megavirales* members other than giant viruses of amoeba, and might share a common ancestor or have been exchanged through horizontal gene transfer with proteins from cellular organisms. Moreover, with our siRNA-based strategy, we are able to functionally annotate 30 proteins from mimiviruses, as well as re-annotate 108 proteins from metagenomic (dark matter) databases and approximately 1,000 hypothetical proteins archived in public sequence databases and encoded by archaea, bacteria, and eukaryotes (**Table 5**).

## Discussion

We demonstrated, using siRNA, that four proteins are involved in Mimivirus fiber formation. A disturbance in the expression of one of these proteins significantly altered the size or shape of these fibers, which indicates that these proteins are either elements of the fiber or involved in fiber formation. To our knowledge, this is the first study that described a modification of Mimivirus virions, and that used siRNA to determine the function of a Mimivirus gene.

In this article, we identified, using RNAi, the function of four proteins, including the L725 and L829 proteins with previously unknown function, a putative oxidoreductase (R135) and a TPR-containing protein (R856; Supplementary Figure S10). Comparative genomic analyses indicated that the L725 encoding gene is an ORFan, while R135, L829, and R856 are unique amongst viruses to mimiviruses, but have homologs in amoeba, bacteria, fungi, and metazoa, and might have been exchanged by horizontal gene transfer. It is noteworthy that Mimivirus protein R856 belongs to the TPR superfamily of proteins that were reported to be involved in protein–protein interactions (Das et al., 1998; Blatch and Lassle, 1999; D’Andrea and Regan, 2003; Cortajarena and Regan, 2006), virus–host interactions (Callahan et al., 1998; Jeshtadi et al., 2010), and regulation of virus replication (Lin et al., 2012; Tani et al., 2013). We provide evidence for a new function of this protein in the formation of Mimivirus fibers.

ORFan and un-annotated genes occupy more than 50% of the gene repertoire of Mimivirus. Here, in addition to providing evidence that four proteins are FAPs by siRNA, our new strategy allowed us to re-annotate 30 proteins in mimiviruses and closely related giant viruses that share sequences homology with FAPs. In addition, this strategy opens a way to re-annotate proteins from sequence databases and genomic dark matter, as was the case here for >500 bacterial proteins and approximately 100 proteins from metagenomes, (**Table 5**).

In this work, we targeted genes of Mimivirus fibers that are easy to observe and study by electron microscopy, immunogold, and proteomics. However, our strategy of silencing ORFan genes in giant viruses opens the way to identify the function of their complete gene repertoires. In particular, the proteins of giant viruses of amoeba, like those from other intracellular species, are poorly expressed and difficult to crystallize, making their functional analysis difficult. This proposed approach will lead to the annotation of hundreds of proteins without known function found in public databases and differentiate between junk DNA and truly used genes.

## Author Contributions

HS, PC, BS, and DR conceived and designed the experiments; HS, BS, IP, DR, and PC analyzed the data; HS performed the experiments; and HS, PC and DR wrote the manuscript.

## Conflict of Interest Statement

The authors declare that the research was conducted in the absence of any commercial or financial relationships that could be construed as a potential conflict of interest.
