# Supplementary material for: Identification of giant Mimivirus protein functions using RNA interference
Source: Front Microbiol. 2015 Apr 28;6:345. doi: 10.3389/fmicb.2015.00345 (PMC4412084; doi:10.3389/fmicb.2015.00345)
Supplement: Supplementary file 1 [file Data_Sheet_1.DOC]

**SUPPLEMENTARY INFORMATION**

**Supplemental results**

**Table S1.** The Excel “xls” spreadsheet contains the protein description, reference sequence (RefSeq accession), accession numbers, and full protein and gene names. The “BLASTp 15 aa Vs mimiviruses” spreadsheet includes the results from a BLASTp search of the 15 candidate proteins from a member of *Mimiviridae* against the entire proteome. “BLASTp of 6 prot vs nr” contains results from a BLASTp search of the four proteins involved in fiber formation and additionally two proteins that are conserved within *Mimiviridae* family against the NCBI nr database. “BLASTp of EFC vs Mimivirus aa” contains results from a BLASTp search of poxvirus entry fusion complex (EFC) proteins against Mimivirus proteins. “Blastp fiber protein vs UniParc” contains results from a BLASTp search of six Mimivirus proteins against UniParc database.

**Table S2.** The double-stranded RNA sequences for the tested Mimivirus genes.

| **Gene** |  | **Sense** | **Anti-sense** | **Position** |
| --- | --- | --- | --- | --- |
| R135 | 1 | GCUGUUGUGGAUCGAAUUATT | UAAUUCGAUCCACAACAGCTT | 936 |
|  | 2 | GCUACUUAUGGAGCAAAUUTT | AAUUUGCUCCAUAAGUAGCTT | 1752 |
| L725 | 1 | UCUCCUUGAUGUGAAGUAUTT | AUACUUCACAUCAAGGAGATT | 138 |
|  | 2 | GGUGUUCACAAGGGAAAUATT | UAUUUCCCUUGUGAACACCTT | 592 |
| R829 | 1 | CCACAUCACAAUUUGGUAATT | UUACCAAAUUGUGAUGUGGTT | 575 |
|  | 2 | GCGGCCAAUUAUAGAGAAATT | UUUCUCUAUAAUUGGCCGCTT | 1087 |
| R856 | 1 | GCUUUAGGCGACUAUGAUATT | UAUCAUAGUCGCCUAAAGCTT | 264 |
|  | 2 | CCAUAUUGAAACCGCAUUUTT | AAAUGCGGUUUCAAUAUGGTT | 467 |
| L425 | 1 | GGUCACGUUGAAUUUGCUUTT | AAGCAAAUUCAACGUGACCTT | 265 |
|  | 2 | CCAAGAACCUGGAGGUUAUTT | AUAACCUCCAGGUUCUUGGTT | 993 |

1 and 2 are siRNA duplex.

**Table S3.** Sequences of the PCR primers and probes used for the tested Mimivirus genes.

| **Gene** | **Forward** | **Reverse** | **Probe** |
| --- | --- | --- | --- |
| R135 | 5'-TGCAAACCAATTCCGTGTAA-3' | 5'-AAATGACTATGTCCAGCTTCCA-3' | 5'-CCCACACCAATTCCATGTGATCCC-3' |
| L136 | 5'-TCCATCCTCATCAATATCTACA-3' | 5'-GGAACAAGTGCATTACATGCA-3' | 5'-TTGAGTTCTCGTCCAAGTTGGCG-3' |
| R139 | 5'-CAAACCTGATCCAGAAAGAAAA-3' | 5'-CGATCTAGCCAAATTTAATCCA-3' | 5'-GCTGGTGCAAGCGCCATGAA-3' |
| L142 | 5'-TTTCCTTTTCCAATAACAACACT-3' | 5'-AATCTGAGCCATTTTTTTGTACA-3' | 5'-TCCATTCAACATTCCCAACTGTTTG-3' |
| R641 | 5'-AAATAATATCCACAAGCTTCCAA-3' | 5'-TCCAGAATTAGATTTCAAGATTG-3' | 5'-AATCCACCAGTCAATCCAGATTCAA-3' |
| L725 | 5'-TGGTGCAACTTATCTTCGATCA-3' | 5'-CAAGGAGACGATGGTTGACA-3' | 5'-TTGGCCGCGAAAGATGAATGG-3' |
| L829 | 5'-CTATAAAGACGCTTTCCCGTT-3' | 5'-GAAGCTAACCTAAGATCATCAT-3' | 5'-CGAAACAGAAATGTCCGGAGTGC-3' |
| R856 | 5'-GCATTAATGGGGATTGCTTCA-3' | 5'-CAAATGCGGTTTCAATATGGTT-3' | 5'-AAGGAAATTACGATGAGGCACTCTC-3' |
| mg878 | 5'-AGGCTGGTTCATGGCATTTA-3' | 5'-CAACACCCATACCATGAGCA-3' | 5'-TGGCAAGCACCTCTCCAACCTG-3' |

**SUPPLEMENTARY FIGURES**

**Fig. S1**. Representative figure for wild type virus (WT) with normal, stretched and long fibers that form a dense layer, which is used as experimental control.

**
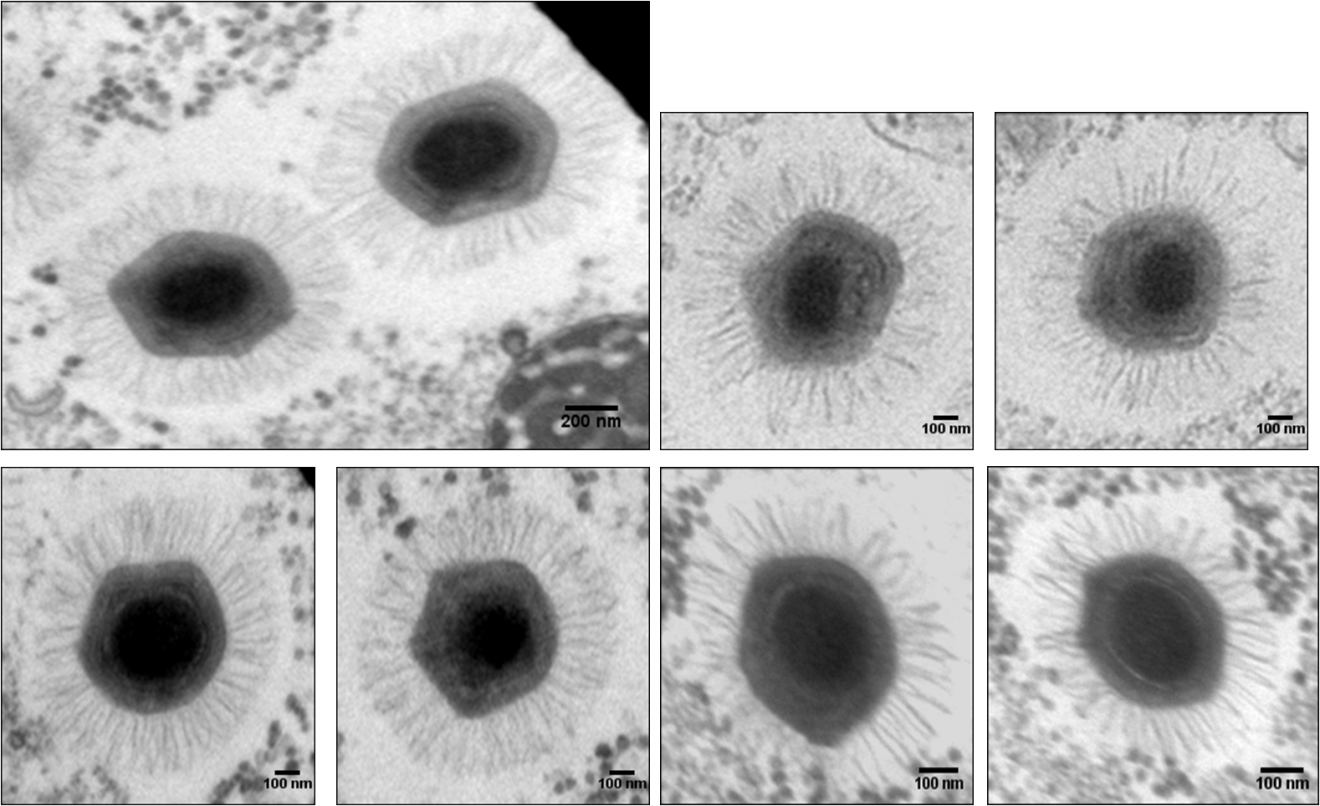
**

The average length is approximately 130 nm/fiber.

**Fig. S2**. Representative figure for shape of the fibers after silencing the L425 capsid protein, control virus.

**
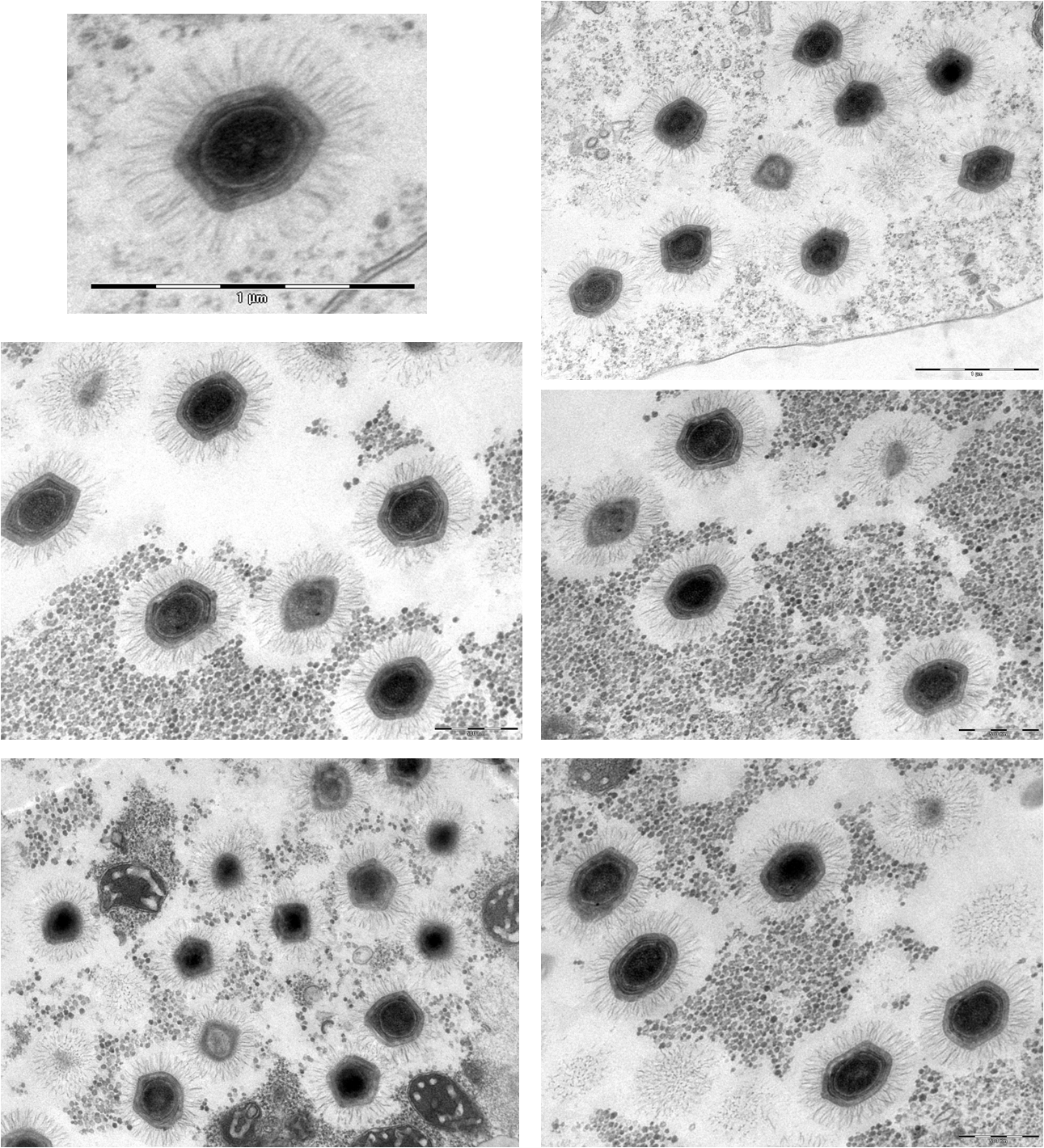
**

**Fig. S3**. Representative figure for shape of the fibers after silencing the R856 protein.

**
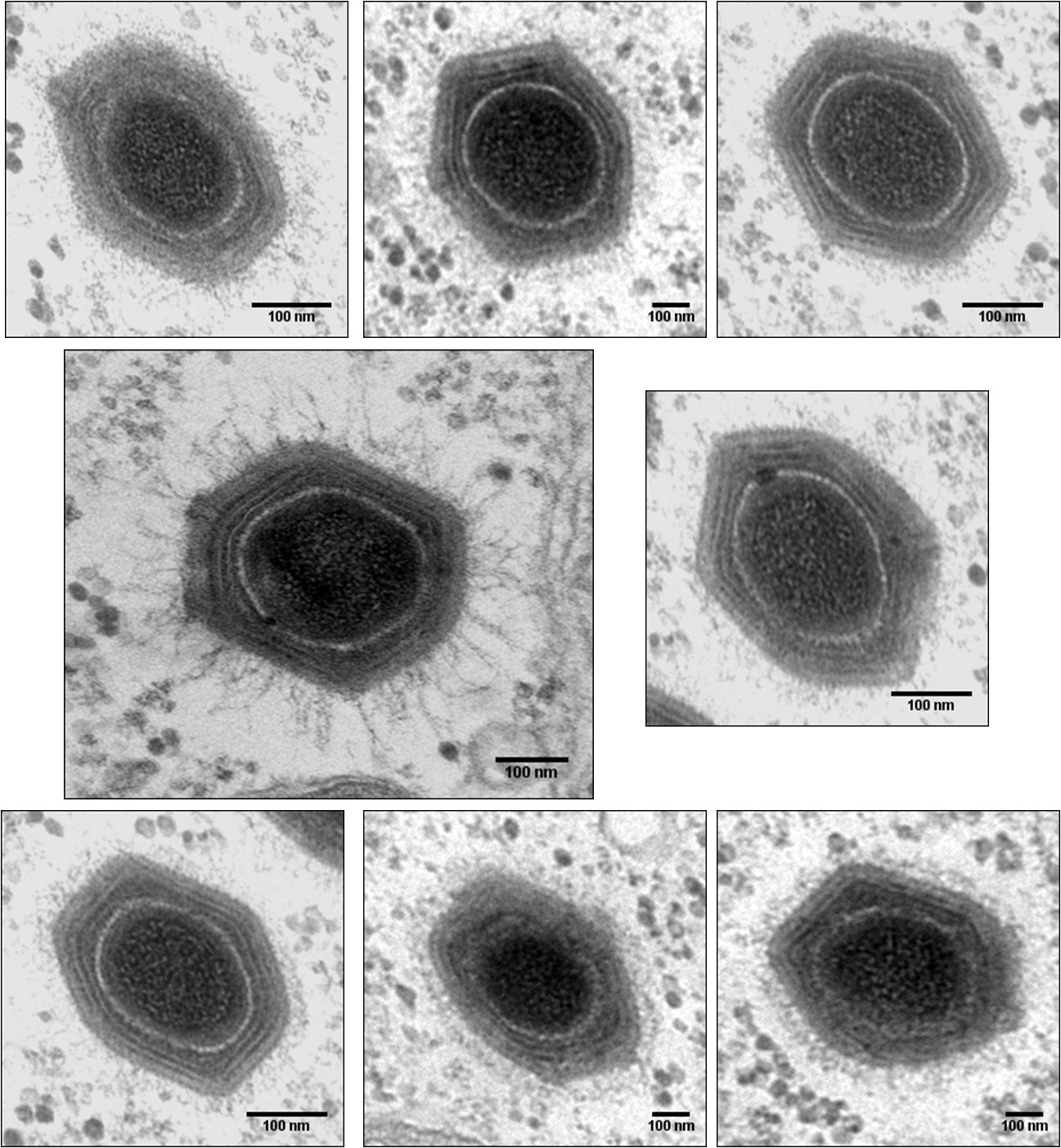
**

The fibers are very short (<50 nm / fiber) and only 30% of the normal length of fibers.

**Fig. S4**. Representative figure for shape of the fibers after silencing the L725 protein.

**
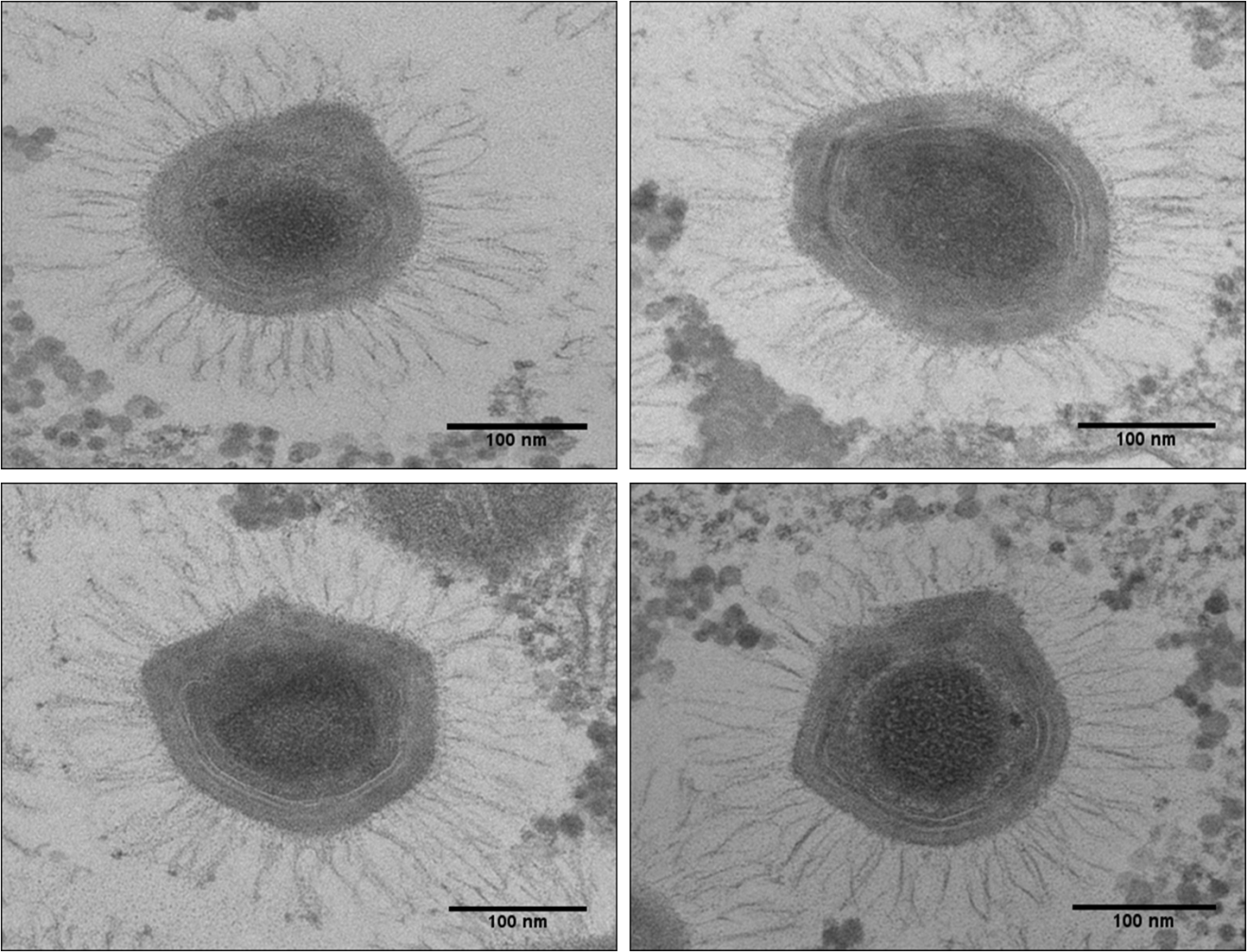
**

The fiber layer is less dense, and the fibers are curved and shorter than those of the normal virus by approximately 15%.

**Fig. S5**. Representative figure for shape of the fibers after silencing the L829 protein.

The fiber layer is less dense. The fibers are curved, not stretched, and shorter than those of the normal virus by approximately 30%.

**Fig. S6**. Representative figure for shape of the fibers after silencing the R135 protein.

**
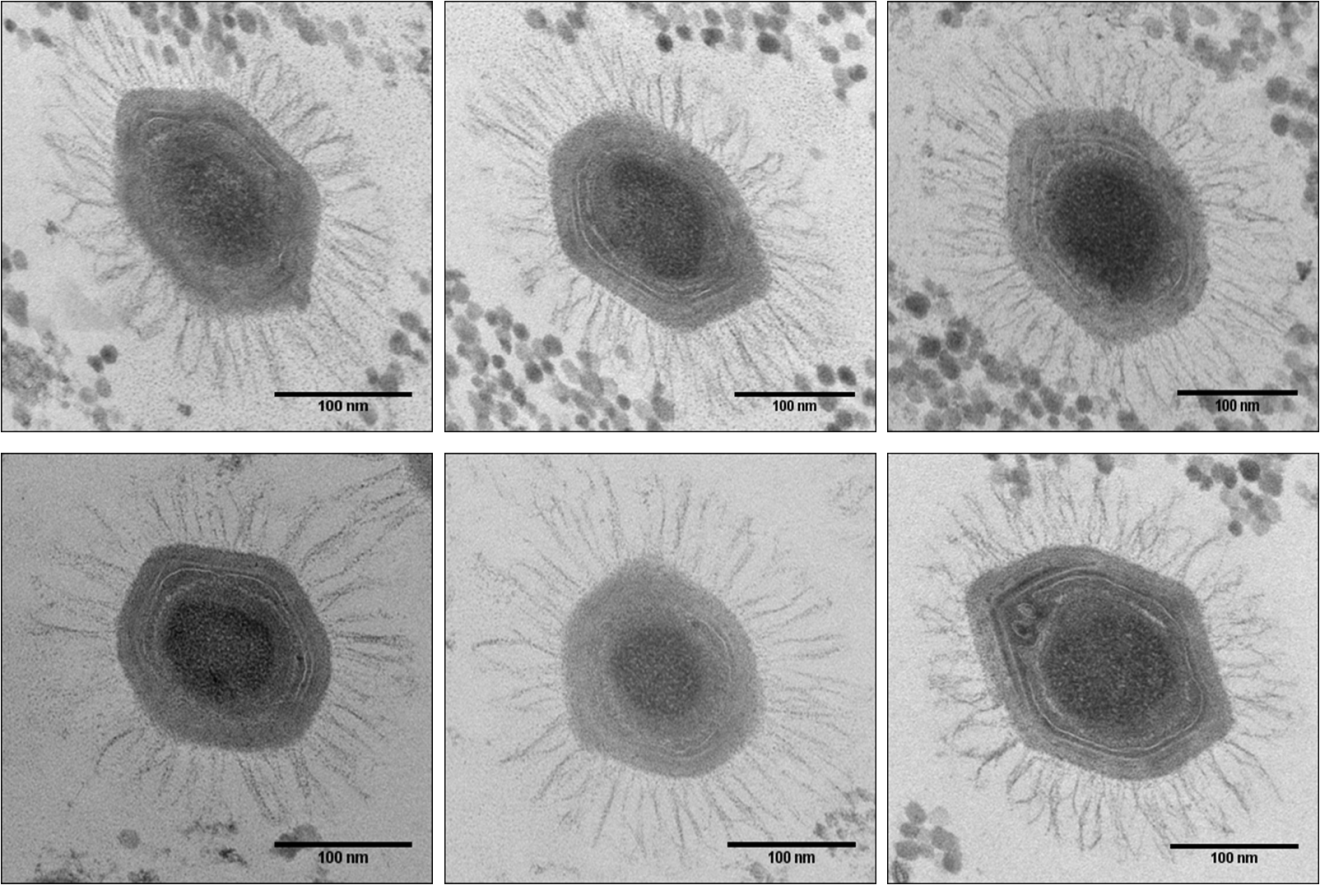
**

The fiber layer is less dense, and the fibers are curved and shorter than those of the normal virus by approximately 12%.

**Fig. S7**. Electron micrograph showing fiber shape, structure, and thickness in mimiviruses.

**
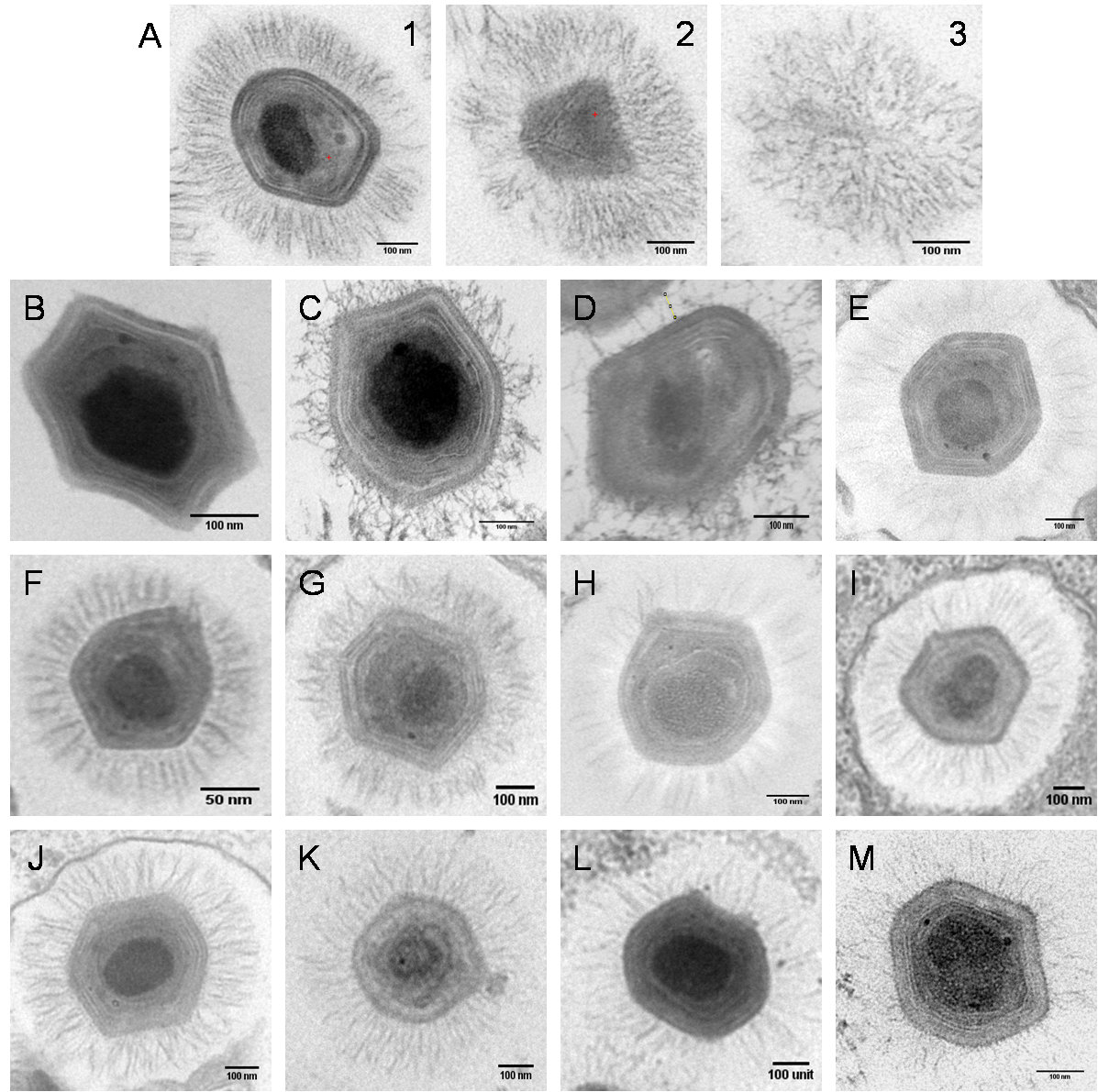
**

Mimivirus has a large and dense layer of fibers; the M4 strain has no fibers; Mamavirus and Lentillevirus have a short and thin layer of fibers.

A: Mimivirus (1: transverse section of the virion; 2: star-shaped capsid vertex with no fiber; 3: top view of one of the capsid vertices showing the fibers); B: Mimivirus M4 strain; C: Mamavirus; D: Lentillevirus; E: Montpellier virus 3; F: Courdo7 virus; G: Courdo11 virus; H: Terra1 virus; I: Terra2 virus; J: Pointe-rouge1 virus; K: Pointe-rouge2 virus; L: Longchamp virus; M: Lactours2 virus.

**Fig. S8**. Phylogenetic tree reconstruction using Maximum likelihood method for R135 protein (FAP1).

Viruses, bacteria and eukaryotes are shown in red, black and blue, respectively. The percentage of replicate trees (100 replicates) in which the associated taxa clustered together in the bootstrap test are shown next to the branches. The number represents the bootstrapping values and the scale bar represents the number of estimated changes per position for a unit of branch length

.

**Fig. S9**. Phylogenetic tree reconstruction using Maximum likelihood method for L829 protein (FAP3).

See legend for figure S8.

**Fig. S10**. Phylogenetic tree reconstruction using Maximum likelihood method for R856 protein (FAP4).

See legend for figure S8.
